# Using calcaneal plates in fixation of comminuted posterior wall acetabular fractures with cranial or posterior extension: a prospective case series and novel technique

**DOI:** 10.1007/s00590-024-03939-1

**Published:** 2024-04-20

**Authors:** Mahmoud Fahmy, Ebeed Yasin, Mohamed Abdelmoneim

**Affiliations:** 1 Pelvis Fracture and Arthroplasty Unit, orthopedic Department, Kasr Alainy Hospital, Cairo University, Cairo, Egypt; 2 orthopedic Department, Aswan University, Aswan, Egypt; 3 Pelvis Fracture and Arthroplasty Unit, orthopedic Department, Kasr Alainy Hospital, Cairo University, Cairo, Egypt

**Keywords:** Calcaneal plate, Fixation, Posterior wall

## Abstract

**Background:**

This study aims to evaluate the outcomes of using calcaneal plate in fixation of comminuted posterior wall (PW) acetabular fractures especially that have cranial (dome) or posterior extension (posterior column edge), and to evaluate its safety. To our knowledge, this is the first study that utilizes this off label implant technique in fixation of such fracture.

**Methods:**

Twenty-two patients enrolled in the study with a minimum follow up of one year. After reducing the PW fragments sequentially, calcaneal plate was applied, fixing its distal part at ischial tuberosity upper ends using 3 screws in a triangular fashion, while its proximal part and radial wings were firmly fixed along the acetabular rim together with the classic longitudinal anchorage. Any fixation failure or head subluxation was recorded.

**Results:**

Radiological outcome showed 18 cases scored as excellent, 2 were good, and 2 were poor. The functional outcome revealed 2 patients were excellent, 6 were very good and 14 were good. There was no loss of reduction or fixation failures throughout the follow up period.

**Conclusion:**

Calcaneal plate may offer an alternative method of fixation of comminuted PW fractures with acceptable radiological and functional results. Our study result may encourage the comprehensibility and replicability of this practice, however randomized multicentered studies should be conducted to validate this assumption. This method provides valuable trick strategy, stable and soft-tissue-friendly fracture fixation where modern implantations may be unavailable or of high cost. Calcaneal plates show some fascinating features that allow using them outside their field being flexible with large footprint area for fracture buttressing beside numerous hole choices with diverse paths providing suitable fixation, articular stability and wide zone of coverage in PW comminuted fracture patterns with cranial or posterior extensions. The plate proximal triangular configuration together with distal triangular screw fixation gives a stiff rigid anchorage and buttressing similar to a metal mesh covering and fixing any fragment numbers up to dome level.

## Introduction

Posterior wall (PW) fractures, either isolated or in a comminuted form, represent a major percentage of all acetabular fractures [1, 2]. Restoring good anatomical relationships with rigid fixation for these specific fractures is the key for accepted functional recovery [3]. The residual steps or gaps can increase the local contact pressure of the joint, leading to the occurrence of traumatic arthritis [4].

Therefore, in recent years, researchers have been devoted for assessing different ways of fixation [5–10]. The straight forward policy for fixation of these fractures using lag screws, spring plates, in addition to buttressing reconstruction plates, doesn’t guarantee stable biomechanical stiff construct against fixation loss [7]. Many reports declared fixation failure using the classical fixation method throughout the follow up periods [8]. This may be explained by the fact that PW fractures, with their unique configuration and geometry, may need not only a longitudinal buttressing but also horizontal or radial reinforcement [9]. Due to this challenging fixation issue, many studies suggested other types of rigid construct formation using different implants with different follow up radiological and functional results [10].

The aim of our study was to assess the radiological and functional outcome of using the calcaneal plate in fixation of posterior wall fractures having the comminuted pattern with dome or posterior extension to posterior column edge as a replacement for the fragment specific technique using multiple implants, in addition to safety evaluation and operative time needed.

## Material and methods

From January 2018 to February 2022, a prospective trial was conducted in a level I trauma center in a university hospital after obtaining approval from our institution’s ethical committee. Patients with multi-fragmentary posterior wall fractures above 16 years old were included while neglected, pathological and other types of acetabular fractures were excluded. All patients were preoperatively evaluated clinically and radiologically, consented, and adequately prepared for the operation. All fractures were classified using Judet and Louternal classification. Preoperative data including age, sex, mode of trauma, associated fractures, fracture classification and initial fracture displacement were collected. All surgeries were performed by one of the authors whom are senior pelvis and acetabular fracture consultants.

All patients were operated in the lateral position using Kocher–Langenbeck approach on a radiolucent table under spinal epidural anesthesia. Proper exposure of PW fracture was done using Homan retractors that were applied anteriorly (hammered in the iliac bone) and laterally gently, through the lesser and occasionally the greater sciatic notch, to allow proper identification of the whole posterior wall bed.

Dissection was limited distally to the root (upper end) of the ischial tuberosity, without the need for its full exposure, as preparation for plate application. Blood clots and debris were removed, followed by gentle axial distraction of the hip joint, to allow for saline irrigation of the joint and removal of any intra articular particulates.

**Fig. 1:**
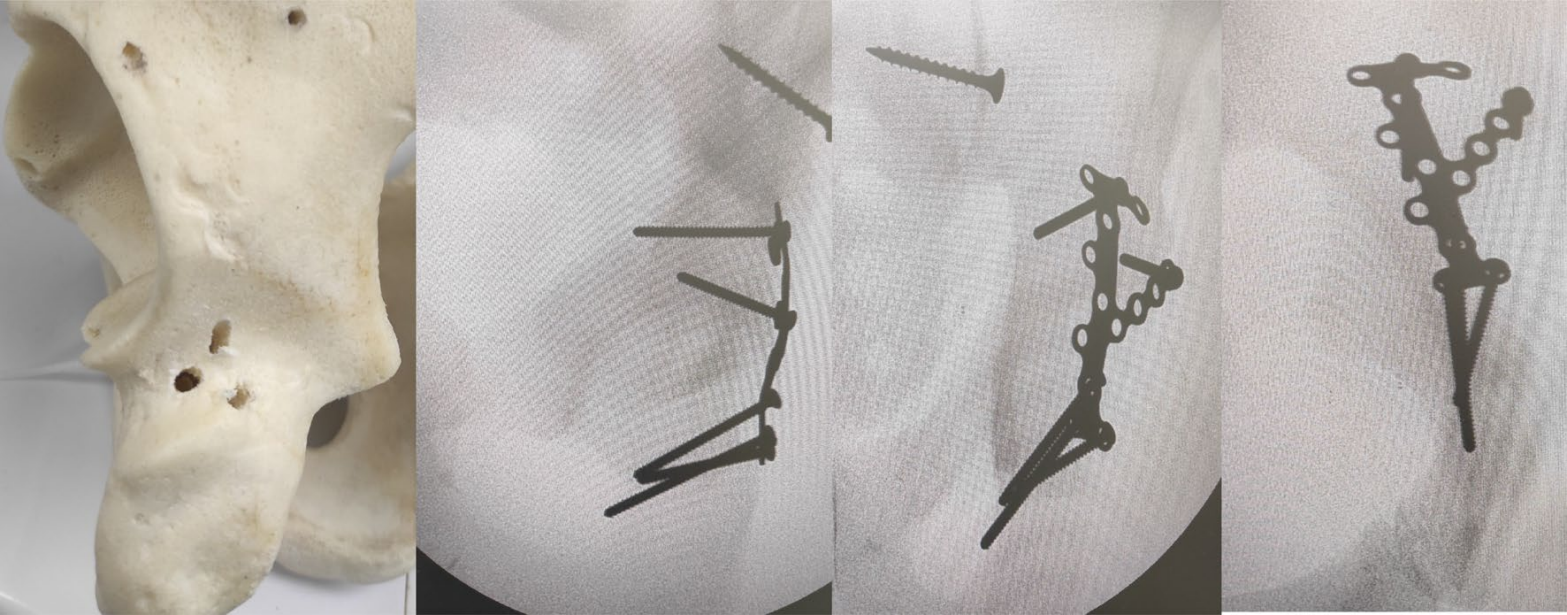
Saw bone and its radiograph demonstration showing 3 ischial screws can be inserted in a triangular pattern at the ischial tuberosity upper end together with the V shape anchorage of the proximal part of the plate

**Fig. 2:**
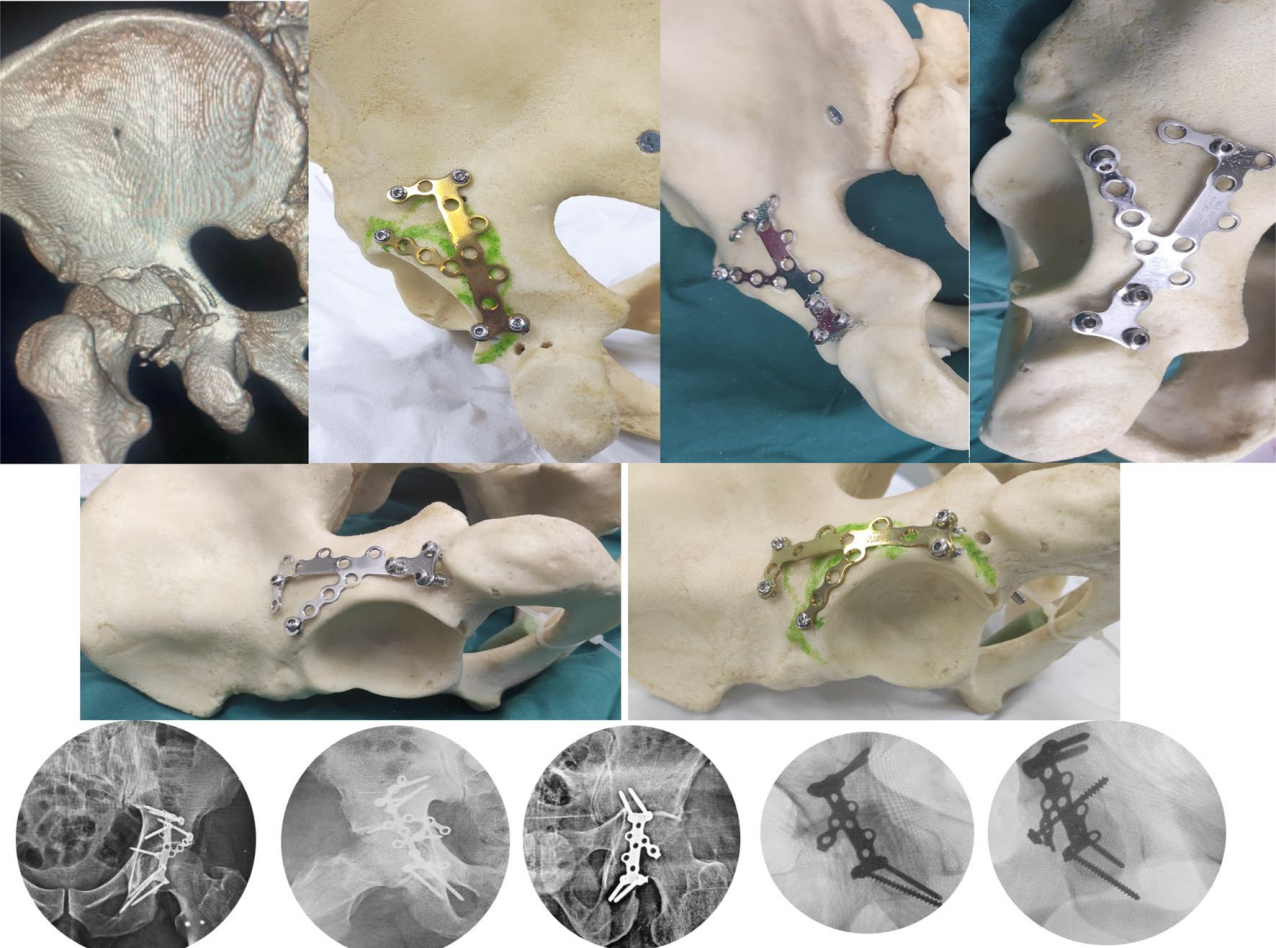
Saw bone demonstration showing calcaneal plate application with large footprint area to cover all expected posterior wall fragments and case examples radiograph

Reduction of the fragments was done accurately seeking an anatomical reduction, starting from the superolateral position then distally around the acetabular rim, using two ball spike pushers gently at both fragment ends. After achieving satisfactory reduction, preliminary fixation using small K wires (0.9 mm diameter) for each fragment similar to the fragment specific fixation technique, however single implant will be used instead of multiple ones,

A suitable size and shape AO titanium calcaneal plate (DePuy Synthes) was then selected according to the location and size of the PW fragments that can fit easily thorough the posterior column corridor being slim of low-profile state. The lower end of the plate applies to the upper end of ischial tuberosity, with no need for further dissection of all soft tissues down to the ischial tuberosity lower end. Three screws were then applied easily in the ischium pillar in a triangular pattern with proper direction and purchase (Fig. 1). This was followed by proper contouring of both the longitudinal and the radial wings of the plate, ensuring full coverage of all the posterior wall area, fixing every fragment specifically through one or more screw. The slim low profile plate wing can be applied very closely to the superolateral acetabular rim in case of presence of very small PW fragment alone or with labral detachment (osteochondral fragments), ensuring adequate buttressing and fixation (Fig. 2).

Fluoroscopic views (AP, Obturator and iliac) were done to confirm the anatomical reduction, screw placement accuracy and trajectory away from joint penetration. Operative time, blood loss, intra and postoperative complications were recorded.

Postoperatively, a rehabilitation program was conducted, allowing partial weight bearing ambulation after six weeks, moving to full weight-bearing after complete healing.

Radiologically, patients were evaluated using Matta criteria, with immediate postoperative CT and post-operative radiographs (AP and Judet oblique views), that were also performed at 2 weeks, 6 weeks, 12 weeks, 6 months, and 12 months, to assess any loss of reduction, residual fracture displacement, head subluxation, fixation failure as well as screw locations and to record complications such as avascular necrosis (AVN), and post-traumatic arthritis. Functionally, patients were evaluated using Merle d’Aubigné and Postel score at the final follow up visit, with an average follow up period of 12 months.

**Fig. 3:**
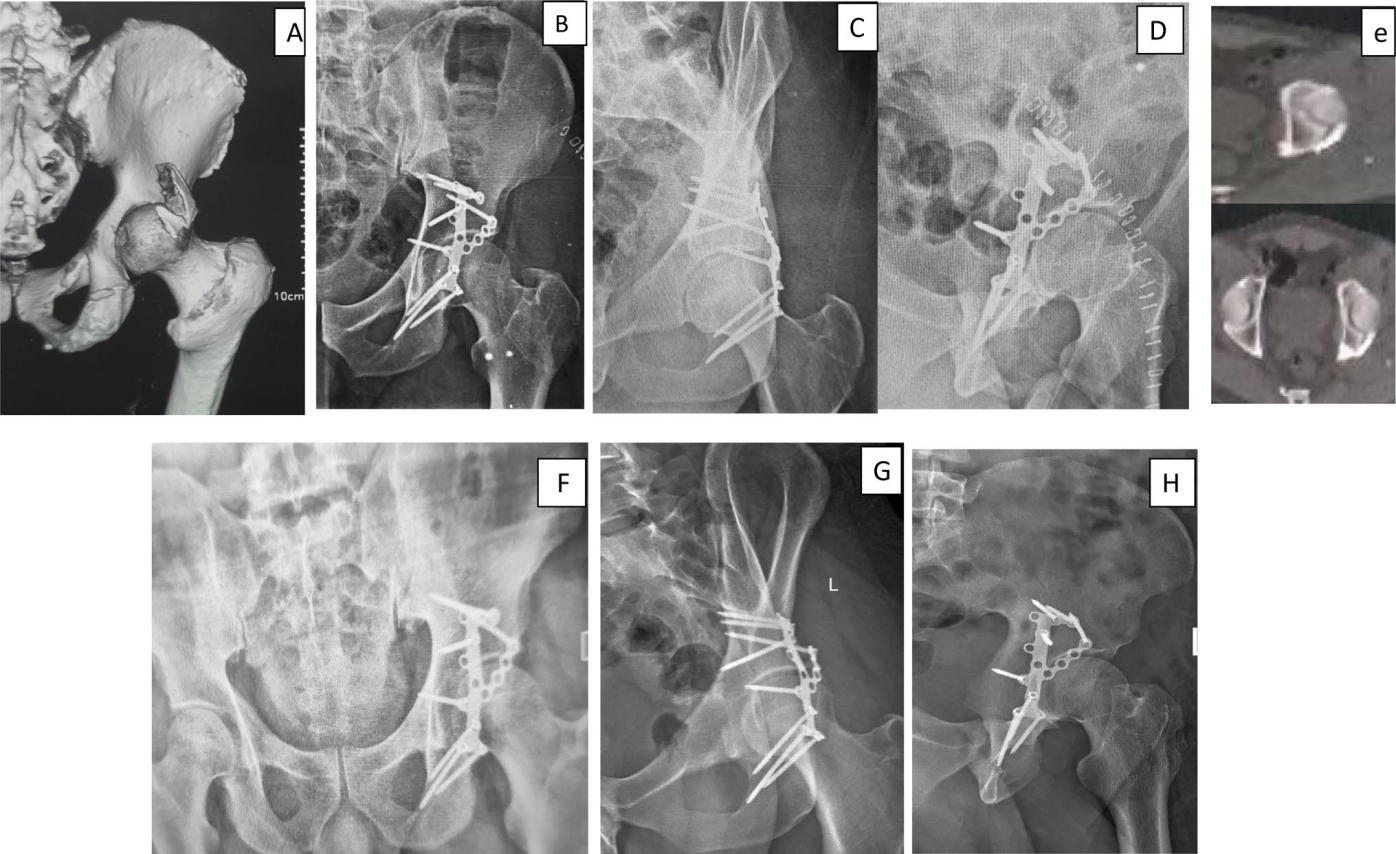
**A** 3D CT scan of 41 year-old male patient showing comminuted PW fracture of the left hip. **B**, **C**, **D** Postoperative radiograph (AP and Judet views) after calcaneal plate fixation showing anatomically reduced fracture. **E** Postoperative CT (axial cuts) after fixation showing anatomical reduction. **F**, **G**, **H** 1 year radiographs (AP and Judet views) show stable fixation with no reduction loss or fixation failure

**Fig. 4:**
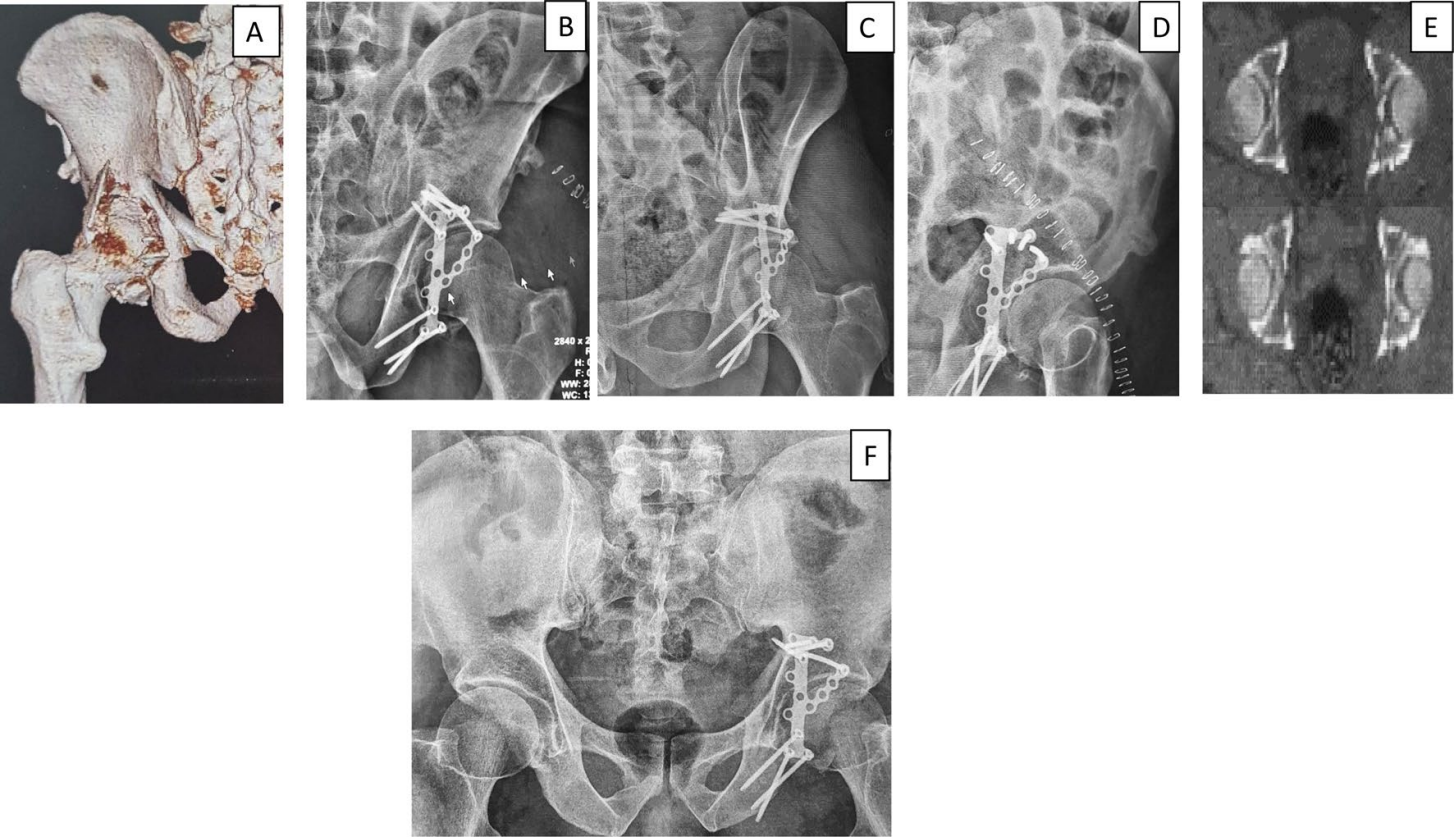
**A** 3D CT scan of 46 year-old male patient showing comminuted PW fracture of the left hip. **B**, **C**, **D** Postoperative radiograph (AP and Judet views) after calcaneal plate fixation showing anatomically reduced fracture. **E** CT (axial cuts) after fixation showing anatomical reduction. **F** 1 year radiographs show stable fixation with no reduction loss or fixation failure

Statistics were coded and processed using the statistical bundle for the Social Sciences (SPSS) version 26 (IBM Corp., Armonk, NY, USA). Evaluations between quantitative variables were completed by means of the non-parametric Kruskal–Wallis and Mann–Whitney tests (Figs. 3, and 4).

## Results

During the duration of our study, we received 33 cases of comminuted PW fractures, 6 cases were excluded due to its association with transverse fracture and 5 cases were also associated with posterior column and omitted. Only 22 cases with comminuted PW fractures (more than 2 fragments) met our inclusion criteria. Posterior hip dislocation or subluxation at the initial time of injury was recorded in 20 patients and was reduced on emergency basis within 6 h. Preoperative neurovascular examination showed 3 cases with sciatic nerve palsy who were resolved at the final follow up visit. There were no associated fractures recorded except in 4 patients who had upper limb fractures or lumbar vertebrae fractures.

Sixteen males and 6 females were included, with a mean age of 42.3 ± 8.6 years (range: 19–51 years). The mode of trauma was motor car accident (MCA) with dash bord injury in 17 patients, falling from height (FFH) in 4 patients, and motor bike accidents (MBA) in 1 patient. The mean time between the date of trauma and surgery was 4.2 days (range: 2–7 days).

Marginal impaction was recorded in 2 cases and treated using elevation and bone graft, while femoral head impaction was encountered in one case. No cases needed trochanteric osteotomy or extensile approaches. The mean operative time was 60.7 min (range: 45–75 min). The mean estimated blood was 600 ml (range: 450–650 ml).

Regarding implant used, traditional non locked AO calcaneal plates were used in 16 patients, and locked calcaneal plates in 6 patients, based on the bone quality and degree of comminution.

Anatomical reduction was achieved in 20 patients, while imperfect reduction was recorded in 2 patients according to Matta’s criteria. Radiological outcomes were evaluated at the final follow up visit, showing 18 cases scored as excellent, 2 cases as good, 2 cases as poor results. The mean follows up period was 20.5 months ± 1.6 (range: 12–28 months).

The functional outcome was evaluated using Merle d’Aubigné and Postel score, where the mean score was 13 (range: 11–18) at the final follow up visit. Two patients were graded as excellent, 6 patients were graded as very good, and 14 patients were graded as good.

All cases showed fracture union with a mean union time of 2.5 months (range: 2–3 months). There was no loss of reduction, fixation failures or heterotopic ossification. Two patients developed late radiological posttraumatic arthritis and were scheduled for total hip replacement after exclusion of infection.

Two cases showed wound infection that improved by debridement and retention of implant. No iatrogenic sciatic nerve palsy was reported postoperatively. The relation between different factors among our study groups, including relationship between age of patients and time needed for union, showed no statistically significant correlations. In addition, no statistically significant correlations were found between age and functional outcome scores at the 1 year follow up.

## Discussion

The gold standard rule for better functional recovery for challenging PW comminuted fractures is anatomical reduction and stable fixation with early rehabilitation [11]. However, outcomes in literature were unsatisfactory [12]. This can be attributed to multiple confounding variables such as dislocation reduction delay, post traumatic arthritis or avascular necrosis [13]. Thus, accepted reduction and fixation may not secure the best results [14].

Multiple fixation methods were described for fixation of PW comminution [15–20]. Reconstruction plates with lag screw fixation from the ischium to the ilium still have the upper hand, and are considered the classical routine plan [16]. This method needs extensive soft tissue and muscle dissection to reach the inferior ischial pole, passing the whole posterior column length to ensure efficient number of screw fixation above the displaced fragments. In addition, contouring difficulties could be encountered, especially if the fragments’ location extends to the superior acetabular portion away from the main posterior pillar, which limits the ability of proper strong buttress fixation against the dislocation force [16–18]. These difficulties could be overcome using our different fixation and implanting technique.

Other options were suggested to facilitate the accessibility of the fragment’s fixation, including one third tubular plate, T plates, cervical plates, and Pilon pates without the need for ischial plate anchorage [18–21]. These options focused on fixing every fragment specifically with full contact buttress contoured plate [21]. Peripheral proper bending and contouring are still points of controversy, and there are no biomechanical and clinical studies focusing on posterior wall fragment specific fixation [20.21]. Anatomical plates were described, but are of high cost and are difficult to apply in variable anatomical shapes with narrow posterior column corridors [20]. Our different technique bypassed most of these obstacles.

The anatomy of the PW is not a longitudinal pillar of bone like the posterior column; however, it has a curved circular shape with tapered edges till it meets the dome anteriorly and completes as the anterior wall [15]. This peculiar shape can’t be fixed with just a longitudinal implant, especially in multiple fracture lines reaching the dome, and needs another horizontal radial plane along the acetabular rim [16, 20].

This could be achieved using multiple separate plates as mentioned in some reports [16], or by using a single versatile implant like the calcaneal plate mentioned in our study. To our knowledge, the current study is the first to utilize this plate for comminuted PW fracture. The wide anatomical area of the PW, that has variable trajectories especially over the superior dome, makes it difficult for a single plate to be applied, and needs a wide coverage plate with long side holes attached to the main longitudinal one. We found these requirements in the calcaneal plate, which is also relatively affordable and readily available compared to other fixation methods.

We used this plate as two triangular zones, a V-shaped proximal part, and a distal part with a zone that has 3 screws in a triangular arrangement. The ability of the calcaneal plate to buttress lateral wall blow out in calcaneal fracture resisting the axial load of ankle joint may resemble its ability to buttress PW fractures resisting dislocating hip force. These plates gained popularity in the fixation of pelvic and acetabular fractures as mentioned in some studies. However, they have not been previously used to fix multi-fragmentary PW fractures as a case series [22, 23]

This new idea regarding -PW fragment specific fixation using a calcaneal plate -appeared to be applicable with multiple advantages. It has a low-profile outfit, which could be easily contoured without extensive dissection. It doesn’t necessarily reach the lowest point of the ischium for fixation. It has multiple radial wings that could be molded easily over the superior acetabular dome in radial position without losing the longitudinal formal frame direction. In addition, multiple lag screws could be applied from these multiple holed plates.

This plate has a large malleable footprint area for either specific fragment fixation or buttressing the whole fragment while applying screws in diverse trajectory planes that can meet anatomical variations or narrow posterior column corridors.

Being a cost-effective option compared to modern expensive implants, it could be a valid fixation strategy in developing countries. Cho et al. used a 3 D printer-assisted plate precontouring technique with a locking screw option to bypass the technical efforts of applying longitudinal implants, which is not always accessible and carries possible postoperative complications [20]. We reached a comparable result without using a specific high-cost implant, using a common, cheap, and available plate in orthopedic practice.

Different shapes and sizes of calcaneal plates are available with variable radial wings. The superior portion of the plate could be cut if it is not necessary to go high with the plate. This allows a large versatility to our technique and can meet almost all intraoperative needs for fixing one or more fragments, whatever the size and location. In case locked screws with the angular stability of locking plates are used, a more stable biomechanical construct can be achieved.

We believe that such plates act as a buttress mesh over comminuted fragments with multiple anchorage points in different longitudinal and radial directions, without the need for full dissection of the ischial tuberosity hump, as it can rest easily on its proximal end with screw trajectories inside the tuberosity ensuring stable fixation. This was reflected in our results where we did not encounter heterotopic ossification, intraoperative neurovascular insult, or ischial tuberosity sitting pain or irritation throughout the follow up period.

This slim malleable plate can be oriented circumferentially around the acetabular rim, which could greatly help buttress very small fragments that may be avulsed with labral detachment (osteochondral lesions) without the use of expensive anchors to reach their beds. These outcomes were comparable to other studies using different fixation constructs [20, 21].

We believe that These plates are not only beneficial in comminuted fractures, but could also be used in large fragment fractures that reach the acetabular dome with its large foot print area and screws in different planes yielding stiffer fixation.

In our pilot study, we did not encounter any cases of fixation loss or hip subluxation. Testing the biomechanical strength of this fixation construct was our primary outcome interest, as the functional outcome prognosis may be affected by different factors not related to fixation manner. These results were in harmony with other studies that used different implants for fragment specific fixations. [2, 8, 11, 12, 20, 21]

The limitations of the study included the small number of patients, absence of a comparative fixation group, a short follow up period that may not reflect accurate clinical outcome, in addition to the absence of any previous biomechanical or cadaveric studies that elaborate the biomechanical strength of calcaneal plates against the posterior dislocating force of the femoral head. Also, the calcaneal plate extends superior and may be difficult to utilize under large abductors muscles being traumatic to the soft tissues. It may be beneficial to cut the superior portion of the plate especially if it's not necessary to go that high with the plate. Further large scale multicentered randomized studies should be conducted to validate these results.

## Conclusion

Fixation of PW comminuted fractures using calcaneal plates with radial extensions remain a suitable option for these challenging fractures and a good alternative to the ischio-ilial reconstruction plate**.** This technique is reliable, helpful, cost effective, safe, and easy, with few risks regarding soft tissue dissection or implant irritation at the ischial tuberosity. It needs little dissection plane to the area of ischial tuberosity, reaching only the ischial root with the application of 3 ischial long screws in a triangular manner that anchor the plate properly to the ischial area beside the V shape anchorage of the proximal plate covering and containing the whole PW bed resembling a wide tailored metal mesh. It acts as multiple miniplates in different radial directions connected together in one construct with high versatility that can accommodate any size or site for the PW fracture.

All cases in our study showed full union with no intraoperative neurovascular injuries, no fixation loss or implant failure signifying a good biomechanical strength for such method. Our study showed different fixation technique and its result may encourage the comprehensibility and replicability of this practice. However, large scale comparative randomized studies regarding other fixation methods should be conducted to validate our results.
